# Morphological characterization and genetic diversity of a new microsporidium, *Neoflabelliforma dubium* n. sp. from the adipose tissue of *Diaphanosoma dubium* (Crustacea: Sididae)

**DOI:** 10.3389/fcimb.2023.1125394

**Published:** 2023-01-27

**Authors:** Meiqi Weng, Xintong Zhang, Zhaozhe Xin, Sijia Xue, Qianqian Zhang, Aihua Li, Jinyong Zhang

**Affiliations:** ^1^ The Laboratory of Aquatic Parasitology, School of Marine Science and Engineering, Qingdao Agricultural University, Qingdao, China; ^2^ Key Laboratory of Aquaculture Diseases Control, Ministry of Agriculture and State Key Laboratory of Freshwater Ecology and Biotechnology, Institute of Hydrobiology, Chinese Academy of Sciences, Wuhan, China; ^3^ College of Advanced Agricultural Sciences, University of Chinese Academy of Sciences, Beijing, China; ^4^ Laboratory for Marine Biology and Biotechnology, Pilot National Laboratory for Marine Science and Technology (Qingdao), Qingdao, Shandong, China

**Keywords:** microsporidia, Rpb1, genetic recombination, sexual reproduction, genetic variation

## Abstract

We reported a new microsporidium *Neoflabelliforma dubium* n. sp. from the adipose tissue of *Diaphanosoma dubium* in China. The infected daphnids generally appeared opaque due to the presence of numerous spore aggregates located in the adipose tissue. All developmental stages were in direct contact with the host cell cytoplasm. Multinucleate sporogonial plasmodia developed into uninucleate sporoblasts by rosette-like fashion. Mature spores were pyriform and monokaryotic, measuring 4.02 ± 0.24 (3.63-4.53) µm long and 2.27 ± 0.15 (2.12-2.57) µm wide (N = 40). The polaroplast was bipartite with a tightly packed anterior lamellae and a loosely aligned posterior lamellae. Isofilar polar filament was coiled 9-11 turns and arranged in 2-3 rows. The phylogenetic analysis based on the obtained SSU rDNA sequence indicated that the *N. dubium* n. sp. clustered with the freshwater oligochaete-infecting *N. aurantiae* to form an independent monophyletic group, positioned at the base of Clade 4. In addition, we analyzed the genetic diversity in three *N*. *dubium* n. sp. isolates based on the rDNA (SSU rDNA, ITS and LSU rDNA) and Rpb1 gene. The genetic variation among the rDNA sequences was not distinct, however, high nucleotide diversity could be observed in Rpb1 gene, and a wide variety of Rpb1 haplotypes were identified within each isolate. Genetic recombination detected in the Rpb1 sequences presumes cryptic sexual process occurring in *N*. *dubium* n. sp. Statistical evolutionary analyses further indicated that the purifying selection eliminated mutations in the Rpb1 gene.

## 1 Introduction

Microsporidia are a group of ubiquitous intracellular unicellular eukaryotic parasites that can infect most animal taxa (Cali and Takvorian, 2014). The origin of microsporidia remained enigmatic with a long history, but it is widely accepted that microsporidia are either a basal branch or sister group of fungi (Keeling, 2014; Wijayawardene et al., 2020; Corsaro, 2022). More than 1600 species assigned to about 220 genera have been described worldwide, among which about half genera were reported from aquatic organisms (Stentiford et al., 2013a; Liu et al., 2020). However, the diversity of aquatic microsporidia is still severely underestimated, especially based on evidence from environmental DNA analysis (Dubuffet et al., 2021; Chauvet et al., 2022). Crustaceans are the common hosts for microsporidia, and more than 64 genera have been reported to infect crustaceans, among which about 38 genera were described from zooplankton crustaceans (Stentiford and Dunn, 2014; Vávra et al., 2016; Simakova et al., 2018; Vávra et al., 2018; Vávra et al., 2019; Bass et al., 2021). Interestingly, a recently phylogenetic analysis indicated that *Mitosporidium daphniae* isolated from *Daphnia magna* was positioned at the root of Microsporidia, indicating that ancestral microsporidia probably originated from aquatic environment (Haag et al., 2014). Elucidating the diversity of aquatic microsporidia should facilitate the understanding of evolutionary biology of microsporidia and explore their potential ecological roles.

The genus *Daphnia* represents a major group of freshwater zooplankton that play an important role in aquatic food chains (Marinho et al., 2018; Toyota et al., 2021) and the important host of aquatic microcrustacean-infecting microsporidia. To date, more than 40 microsporidia belonging to 16 genera have been reported from daphnids (Vávra et al., 2017; Simakova et al., 2018; Vávra et al., 2018; Vávra et al., 2019). However, most of the daphnid-infecting microsporidia were reported solely based on morphological and ultrastructural features (Larsson et al., 1996), with a lack of molecular data, which means that the accurate taxonomy of these microsporidia is still unclear due to the unreliability of morphological and ultrastructural features as taxonomic criteria (Stentiford et al., 2013b; Weng et al., 2021a; Liu et al., 2022). In China, only one species, *Agglomerata daphniae* was reported from the hypoderm of *Daphnia magna* (Weng et al., 2020). To further enrich the knowledge of species diversity of aquatic microsporidia in China and figure out the accurate taxonomy of daphnid-infecting microsporidia, we investigated the diversity of daphnid-infecting microsporidia in the middle and lower reaches of the Yangtze River. In present study, a novel daphnid-infecting microsporidian species, *Neoflabelliforma dubium* n. sp. was described with morphological, ultrastructural, and molecular characteristics. The genetic diversity of this microsporidian was also analyzed referring from the sequence comparison of the rDNA and Rpb1 genes.

## 2 Materials and methods

### 2.1 Collection of specimens and microscopical observation

**Figure 1:**
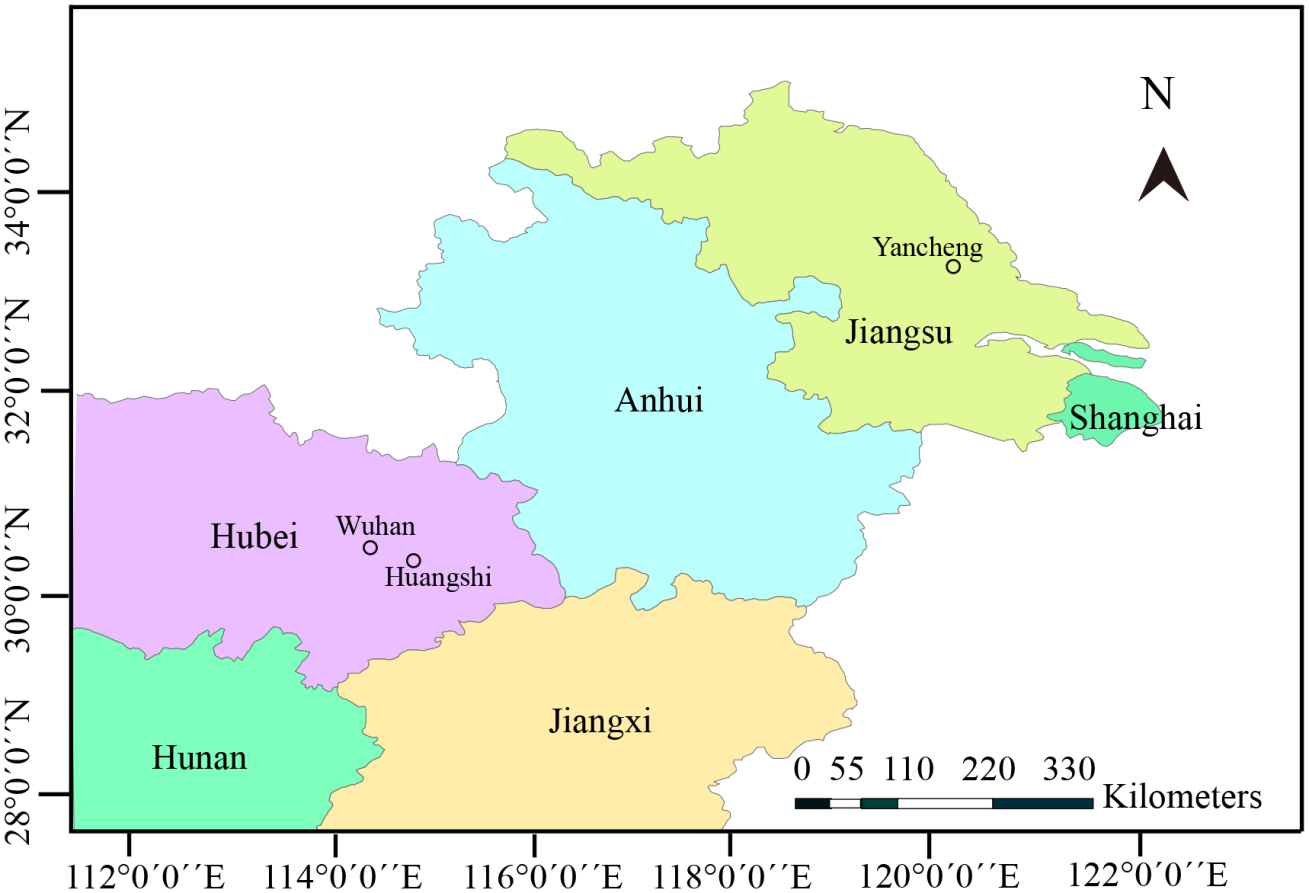
Map of partial China, showing geographical locations of sample sites.

Zooplankton samples were collected from three different locations in the middle and lower reaches of Yangtze River (**Figure 1**). In detail, the samples were collected from a eutrophic pond in Huangshi city, Hubei province (30° 17 ^′^46.49^′′^ N, 114° 44^′^8.53^′′^ E) in June 2019, Yanlong Lake in Yancheng city, Jiangsu province (33° 20^′^0.43^′′^ N, 120° 1^′^39.65^′′^ E) in July 2019, and Jinyin Lake in Wuhan city, Hubei province (30° 38^′^21.35^′′^ N, 114° 11^′^28.64^′′^ E) in October 2020. Specimens were transported immediately to the local laboratory for the preliminary parasitological examination. Daphnids were morphologically identified and screened under Olympus SZ51 microscope. Specimens with opaque coloration were used to make wet mount preparations. Infected cladocerans were preserved in 95% ethanol for further molecular characterization and in 2.5% glutaraldehyde in 0.1 M sodium cacodylate buffer (PH 7.4) for electron microscopic observation, respectively. Formalin-fixed spores were used to capture spore images using an Olympus BX 53 microscope equipped with an Olympus DP72 digital camera (Olympus, Japan). The spores were measured based on capture images by Adobe photoshop CS6 (Adobe System, San Jose, CA, USA), and measurements were presented as Mean ± SD.

### 2.2 Transmission electron microscopy (TEM)

Glutaraldehyde-fixed cladocerans were washed with sodium cacodylate buffer twice. Cladocerans were pos-fixed with 1% osmium tetroxide (OsO4) for 1h, samples were dehydrated in a gradual ascending series of ethanol and propylene oxide, and embedded in Spur resin. Ultrathin sections (70–90 nm) were examined using a Hitachi HT-7700 TEM after being mounted on an uncoated copper grid. Sections were stained with uranyl acetate and lead citrate. Sections of two infected cladocerans were examined using a Hitachi HT-7700 TEM.

### 2.3 DNA extraction, PCR, and sequencing

Ethanol-fixed daphnids were rinsed with distilled water 2 times to get rid of ethanol residues. The daphnids and 400 ml ATL (Qiagen) were added to Lysing Matrix B FastPrep^®^ tubes, which were then homogenized using a FastPrep cell disrupter (2 min at 6.0 m/s). The homogenate was used for genomic DNA extraction using the Qiagen DNeasy Blood & Tissue Kit (Qiagen, Germany) following the manufacturer’s instructions. The primer sets used for rDNA and Rpb1 gene amplification were shown in **Table 1**. PCR was carried out in a 50 μL reaction system, containing PCR buffer, 200 mM dNTP, 2 mM MgCl_2_, 1.25 units Taq polymerase, 20 pmol each primer, and 2 μL DNA template. The partial SSU rDNA was amplified using the primer pair V1f/1492r and the PCR reaction conditions consisted of an initial denaturation step at 95°C for 4 min, followed by 35 cycles at 95°C for 1 min, 50°C for 30s, 72°C for 2 min, and a final extension at 72°C for 10 min. The 3’ terminal partial SSU rDNA, complete ITS and the partial LSU rDNA sequence was amplified using the primer pair HG4F/ILSUR and the PCR cycle included by an initial denaturation step at 95°C for 4 min, followed by 35 cycles of denaturation at 95°C for 30s, annealing at 53°C for 30s, elongation at 72°C for 2 min, and a final extension at 72°C for 10 min. For amplification of the largest subunit of RNA Polymerase II (Rpb1), the primer pair RPB1-F1/RPB1-R1 was used to obtain preliminary sequence which was then used as the template to design specific primers using Primer Premier 5.0. Rpb1 was secondly amplified using the primer pairs NAF/NAR. The amplification was performed under the following conditions: an initial denaturation for 4 min at 94°C, 35 cycles of 30s at 94°C, 30s at 46°C, 1 min at 72°C, and a terminal extension of 10 min at 72°C. The PCR products were excised from an agarose gel and purified using a PCR purification kit (CWBiotech, Beijing, China) and cloned into a PMD-18 T vector system (Takara, Tokyo, Japan). Five positive clones were randomly selected to sequence in both directions with the ABI BigDye Terminator v3.1 Cycle Sequencing Kit and an ABI 3100 Genetic Analyzer.

**Table 1:** The primers used for amplifying and sequencing microsporidia rDNA and Rpb1.

Primer	Sequcence (5’-3’)	References
V1F	CACCAGGTTGATTCTGCCTGAC	(Nilsen, 2000)
1492r	GGTTACCTTGTTACGACTT	(Nilsen, 2000)
HG4F	GCGGCTTAATTTGACTCAAC	(Gatehouse and Malone, 1998)
ILSUR	ACCTGTCTCACGACGGTCTAAAC	(Tsai et al., 2002)
NaRPB1_1F	CG(A/G)AAGTGTGTGTTTTTATTG	(Ironside, 2007)
NaRPB1_3R	GTTTCTGCAGTTTTAATAGCTGTATC	(Ironside, 2007)
NAF	CACCACCAGCAGTGCGACC	Herein
NAR	TCTCCCCAACCAACCTC	Herein

### 2.4 Molecular characterization

The obtained sequences fragments were assembled by BioEdit (Hall, 1999) and the consensus sequences were verified as a microsporidium by a BLAST search. Sequences with high similarity and those of our interest were retrieved from the GenBank database. A total of 65 sequences were aligned with Clustal X by the default setting (Thompson et al., 1997). This alignment was corrected manually using the alignment editor function within MEGA 6.0 (Tamura et al., 2013). *Basidiobolus ranarum* (D29946) and *Conidiobolus coronatus* (AF296753) were used as outgroups. Pairwise genetic distances/similarities were calculated using the Kimura-2 parameter model distance matrix for transitions and transversions. Phylogenetic analysis was conducted using the Bayesian inference (BI) in MrBayes 3.2.4. The optimal evolutionary model was determined to be GTR + I + G by ModelTest 3.7 using Akaike information criteria. Two independent runs were conducted with four chains for one million generations. Phylogenetic trees were sampled every 100 generations. The first 25% of the samples were discarded from the cold chain (burninfrac = 0.25). Tree was initially examined in Figtree v1.4.4 (http://tree.bio.ed.ac.uk/software/figtree/), edited, and annotated in Adobe Illustrator (Adobe System, San Jose, CA, USA).

Nucleotide diversity at synonymous and nonsynonymous sites was estimated by means of the π (Nei, 1987) and *θ*_W_ (Watterson, 1975), applying the Jukes and Cantor correction (Jukes and Cantor, 1969) as implemented in DnaSP 6.0 (Rozas et al., 2017). Tajima’s D (Tajima, 1989) and Fu’s Fs (Fu, 1997) statistics were calculated in DnaSP 6.0 and Arlequin 3.5 (Excoffier and Lischer, 2010), respectively. Genetic differentiation index (Fst) and the corresponding gene flow (Nm) were estimated with DnaSP 6.0. The analysis of molecular variance (AMOVA) (Excoffier et al., 1992) was conducted with Arlequin 3.5. Haplotype networks were performed with Network 10 (https://www.fluxus-engineering.com/sharenet.htm) using the Median Joining (MJ) method.

The recombination events were analyzed with RDP4 (Martin et al., 2015) and SimPlot (Lole et al., 1999). First, the recombination analysis was implemented in the RDP4 by using the available 7 methods (RDP, 3Seq, GENECONV, BoostScan, MaxChi, Chimaera, and SiScan). Default parameters were used. Detected events supported by at least three of the 7 methods were considered as recombination. The recombination events detected by RDP4 were further analyzed in SimPlot. The final recombination events were determined after considering both the SimPlot and RDP4 results.

## 3 Results

### 3.1 Light microscopy

**Figure 2:**
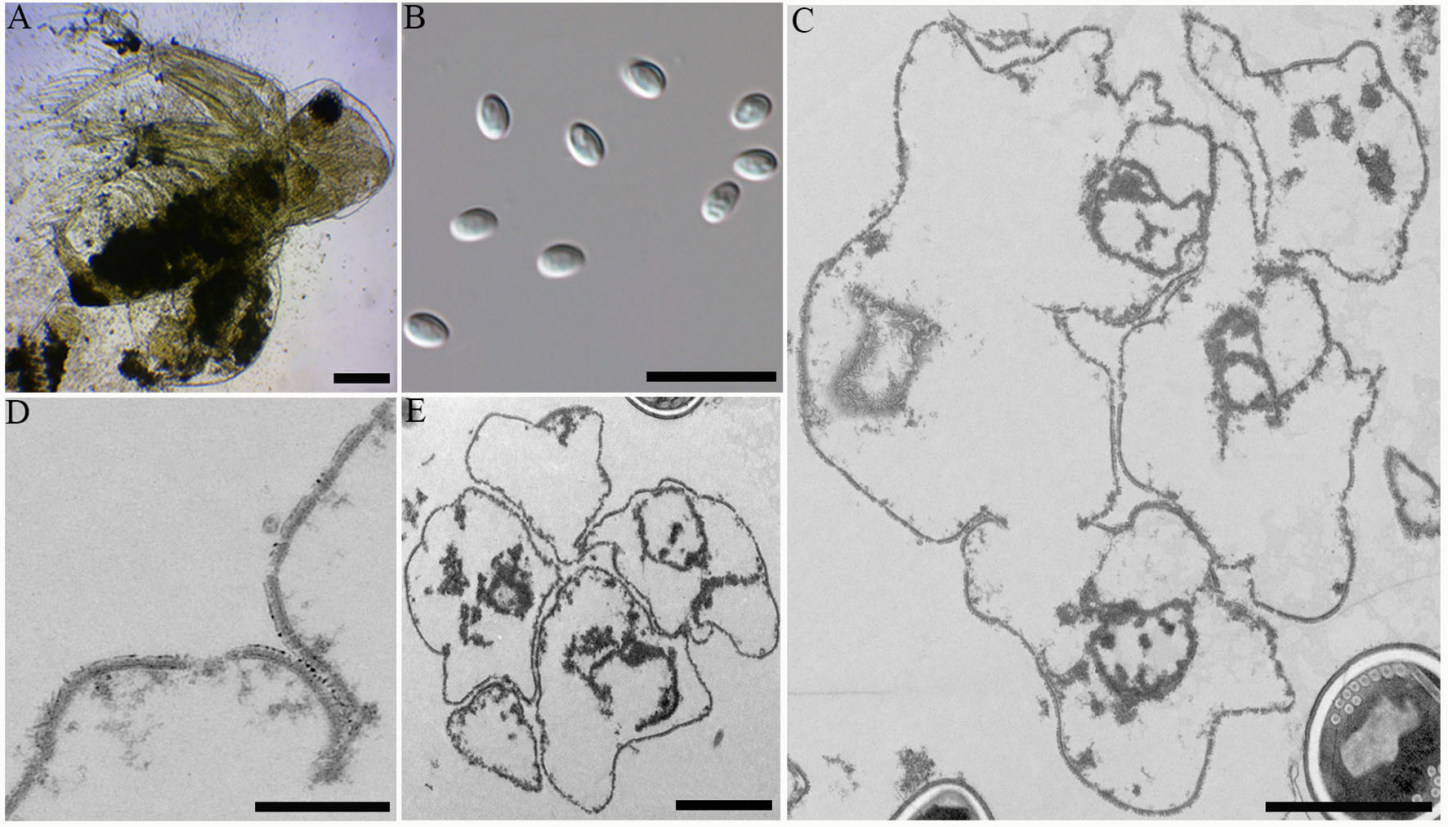
Microscopic observation of *Neoflabelliforma dubium* n. sp. **(A)** Infected *Diaphanosoma dubium* appears opaque, scale bar = 100 μm. **(B)** Fresh spores liberated from infected *D dubium*, scale bar = 10 μm. **(C)** Multinucleate sporogonial plasmodia developed into sporoblasts by rosette-like budding, scale bar = 2 μm. **(D)** The magnification of the electron-dense tubular projections on the cell plasma membrane, scale bar = 1 μm. **(E)** Five irregular sporoblasts were direct contact with the host cell cytoplasm, scale bar = 100 μm.

During sampling, four cladocerans including *Diaphanosoma dubium*, *Daphnia magna*, *Daphnia carinata*, and *Moina micrura* were collected. Among them, microsporidian infection was only found in the adipose tissue of *Diaphanosoma dubium*. Under light microscopy, the infected *D. dubium* generally appeared opaque due to numerous microsporidian spore aggregates distributed in the adipocytes of the host (**Figure 2A**). The prevalence of infection was 1.1% (20/1835) in Huangshi, 2.3% (8/350) in Yancheng and 2.7% (25/925) in Wuhan, respectively. Fresh spores isolated from different locations were oval and the dimension of the spore is identical with 4.02 ± 0.24 (3.63-4.53) µm long and 2.27 ± 0.15 (2.12-2.57) µm wide (N = 40) (**Figure 2B**).

### 3.2 Electron microscopy

**Figure 3:**
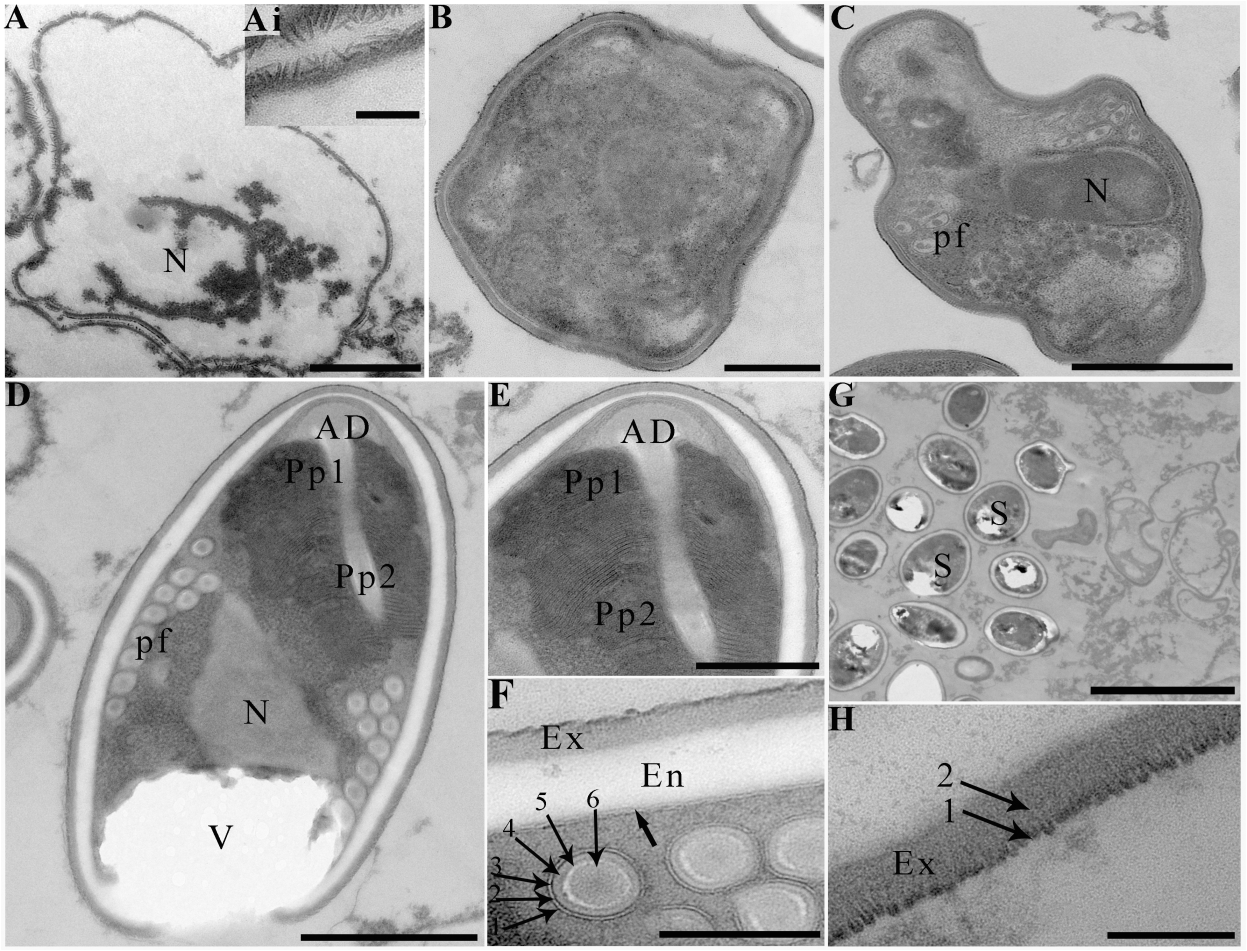
Electron microscopy of *Neoflabelliforma dubium* n. sp. **(A)** An early sporoblasts contained a large nucleus, scale bar = 1 μm. (Ai) The magnification of the electron-dense tubular projections on the surface of sporoblasts, scale bar = 500 nm. **(B)** A sporoblasts with denser cytoplasm, scale bar = 500 nm. **(C)** A late sporoblasts showed the polar filaments and an oval nucleus, scale bar = 1 μm. **(D)** Mature spores contained a mushroom-shaped anchoring disc, isofilar polar filament, a vacuole, a bipartite polaroplast, a large nucleus, and a trilaminar spore wall, scale bar = 1 μm. **(E)** The magnification of bipartite polaroplast showing narrow lamellae and wide lamellae, scale bar = 500 nm. **(F)** Magnification of transverse section of polar filaments showing six discontinuous concentric circles, spore wall including an electron-dense exospore and an electron-translucent endospore, scale bar = 100 nm. **(G)** Mature spores residing in direct contact with the host cell cytoplasm, scale bar = 5 μm. **(H)** Magnification of the exospore showing two distinct layers, scale bar = 100 nm.

Transmission electron microscopy showed that the developmental stages of this microsporidium isolated from different locations were similar. The earliest stages observed were multinucleate sporogonial plasmodia which resided in direct contact with the host cell cytoplasm (**Figure 2C**). Multinucleate sporogonial plasmodia developed into 4-8 uninucleate sporoblasts by rosette-like fission (**Figure 2C**). At this stage, electron-dense tubular projections were visible on the surface of multinucleate sporogonial plasmodia (**Figure 2D**). Early sporoblasts were of irregular shape and possessed a large nucleus and some free ribosomes (**Figure 2E**). Electron-dense tubular projections could be observed on the surface of sporoblasts (**Figures 3A**, 3Ai). In late sporoblast, the cytoplasm was denser, and the precursor of polar filament and exospore were seen (**Figures 3B, C**). Typical spore organelles were observed within the spores, including anchoring disk, polar filaments, polaroplast, the trilaminar spore wall, and posterior vacuole. Mature spores were pyriform and resided in direct contact with the host cell cytoplasm (**Figures 3D, G**). The bipartite polaroplast consisted of a tightly packed anterior lamellae and a loosely aligned posterior lamellae (**Figure 3E**). Isofilar polar filament coiled 9-11 turns and arranged in 2-3 rows. The polar filament measured 112–153 nm in diameter, and exhibited six discontinuous density concentric circles (**Figure 3F**). The spore wall consisted of a 50-59 nm wide layered exospore, and electron-lucent endospore 97-132 nm wide (**Figure 3F**). The exospore contained two layers, including the electron-moderate layer and electron-dense coat of tubular projections (**Figure 3H**). Spore organelles were surrounded by a plasma membrane of 8-12 nm wide.

### 3.3 Molecular characterization

**Figure 4:**
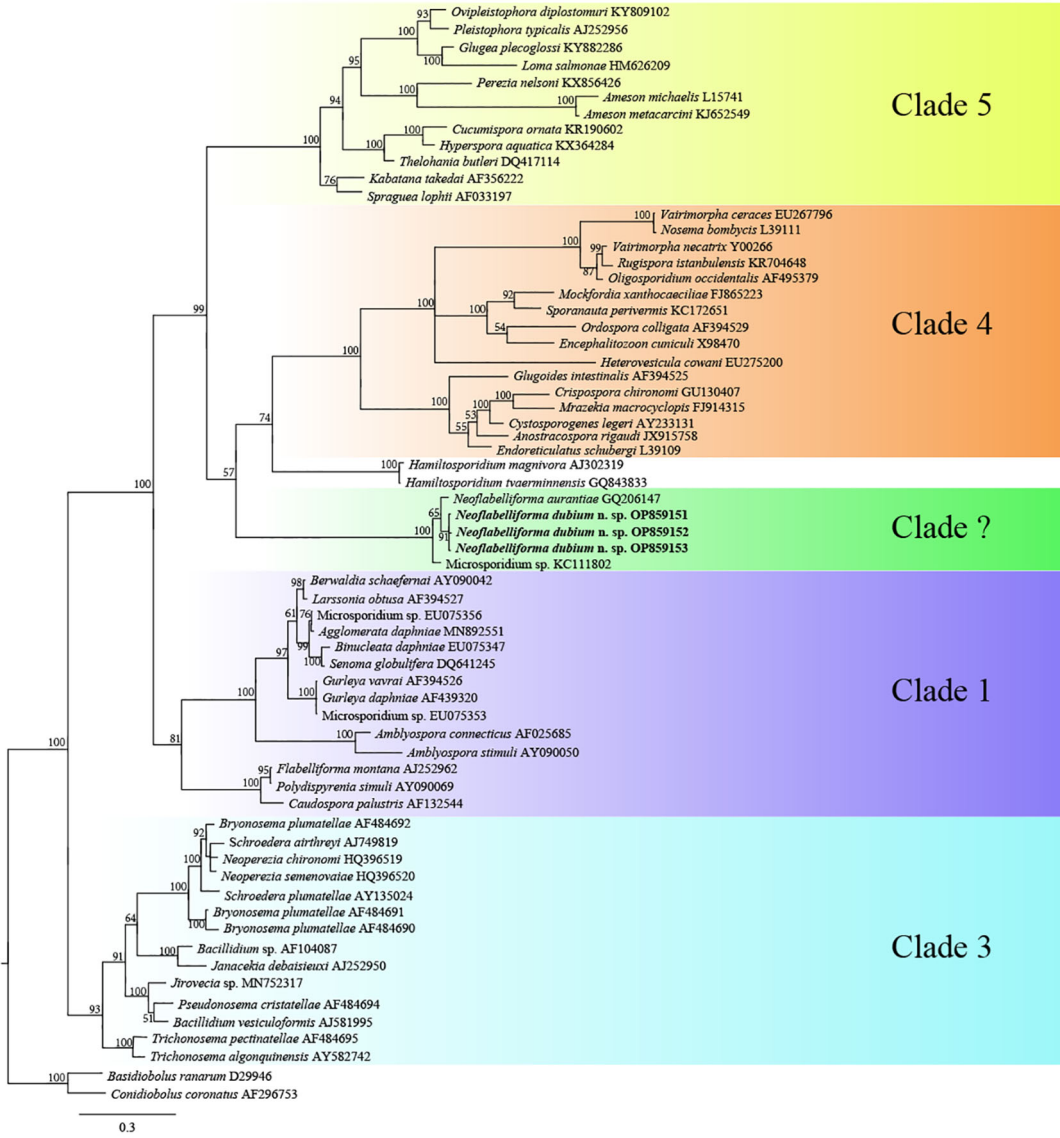
The SSU rDNA-inferred phylogenetic tree of *Neoflabelliforma dubium* n. sp. and the other aligned microsporidian species by Bayesian Inference method. Posterior probabilities were shown on branch nodes. The present species was indicated in bold.

The SSU rDNA sequences of the microsporidia infecting *D. dubium* collected from 3 different locations were deposited in GenBank under accession numbers OP859151-OP859153. Their concatenated partial SSU, complete ITS and partial LSU was assigned with accession numbers OP881373-OP881375. Sequence analysis showed that SSU sequences obtained from the daphnids collected from different locations were of high identity (with 99.41%-99.85% identity) (**Table 2**). Therefore, based on the morphological and high SSU sequence identity, these microsporidia belong to the same species. BLAST searches using the obtained SSU rDNA sequences indicated that the closest relative to our finding was *Neoflabelliforma aurantiae*, a freshwater oligochaete-infecting microsporidium (with 96.42%-96.79% identity). The pairwise distances/similarities calculated by Kimura 2-parameter model between SSU rDNA sequences of this novel and 9 other related microsporidia ranged from 0.0038/99.62% (between the new microsporidium collected from Huangshi OP859151 and the novel microsporidium collected from Wuhan OP859152) to 0.4371/56.29% (between *N. aurantiae* GQ206147 and *Anncaliia meligethi* AY894423) (**Table 3**). To further explore the possible genetic variation among 3 isolates of the novel microsporidium, their full ITS and partial LSU rDNA sequences comparison analysis were performed. Results indicated that the between-isolates genetic variation among the ITS and LSU sequences was not distinct (**Table 2**). The phylogenetic analysis based on the obtained SSU rDNA sequence indicated that these 3 isolates of the novel microsporidium clustered firstly with *N. aurantiae*, and then formed a sister group with an unidentified microsporidium from the soil, which collectively formed a solitary basal branch of the clade 4 (**Figure 4**).

**Table 2:** Percentage of sequence similarity of the rDNA of the *Neoflabelliforma dubium* n. sp. isolated from different geographical locations.

Locations	Gene		
	SSU	ITS	LSU
HS vs WH	99.85	97.83	99.31
HS vs YC	99.41	97.83	99.27
WH vs YC	99.56	100	99.44

HS, Huangshi, WH, Wuhan, YC, Yancheng.

**Table 3:** Pairwise nucleotide sequence identity (upper right) values and evolutionary distances (left bottom) among *Neoflabelliforma dubium* n. sp. isolates and 9 other microsporidium species with high sequence similarity by Kimura-2 Parameter analysis based on SSU rDNA sequences.

Species	1	2	3	4	5	6	7	8	9	10	11	12
1. *Neoflabelliforma dubium* n. sp. OP859151		99.62	99.31	96.40	72.53	69.25	68.12	65.81	64.50	62.32	57.18	56.70
2. *Neoflabelliforma dubium* n. sp. OP859152	0.0038		99.54	96.72	72.66	69.14	68.01	65.97	64.65	62.48	57.58	57.06
3. *Neoflabelliforma dubium* n. sp. OP859153	0.0069	0.0046		96.32	72.66	69.01	67.88	65.97	64.54	62.34	57.16	56.87
4. *Neoflabelliforma aurantiae* GQ206147	0.0360	0.0328	0.0368		72.31	69.35	68.22	66.66	64.12	62.53	56.57	56.29
5. *Naidispora caidianensis* OL583677	0.2747	0.2734	0.2734	0.2769		69.69	68.83	69.80	71.34	70.28	60.08	59.06
6. *Hamiltosporidium tvaerminnensis* GQ843833	0.3075	0.3086	0.3099	0.3065	0.3031		99.01	63.20	63.73	63.96	60.28	59.19
7. *Hamiltosporidium magnivora* AJ302319	0.3188	0.3199	0.3212	0.3178	0.3117	0.0099		62.07	62.77	62.74	59.26	57.80
8. *Bryonosema plumatellae* AF484692	0.3419	0.3403	0.3403	0.3334	0.3020	0.3680	0.3793		79.59	82.02	64.86	62.05
9. *Bacillidium branchilis* ON054959	0.3550	0.3535	0.3546	0.3588	0.2866	0.3627	0.3723	0.2041		84.30	64.75	59.06
10. *Bacillidium vesiculoformis* AJ581995	0.3768	0.3752	0.3766	0.3747	0.2972	0.3604	0.3726	0.1798	0.1570		62.96	62.92
11. *Tubulinosema acridophagus* AF024658	0.4282	0.4242	0.4284	0.4343	0.3992	0.3972	0.4074	0.3514	0.3525	0.3704		73.90
12. *Anncaliia meligethi* AY894423	0.4330	0.4294	0.4313	0.4371	0.4094	0.4081	0.4220	0.3795	0.4094	0.3708	0.2610	

### 3.4 Genetic diversity

**Figure 5:**
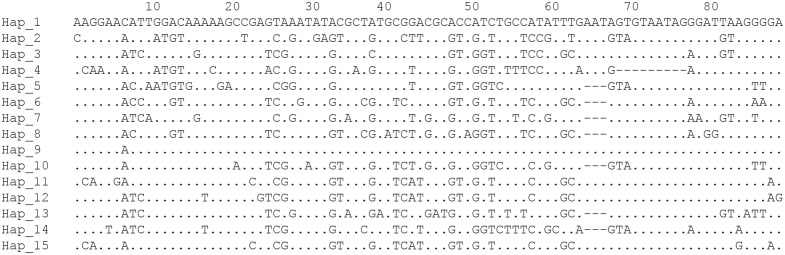
Haplotypes of the Rpb1 fragment of *Neoflabelliforma dubium* n. sp. showing only the polymorphic sites.

**Figure 6:**
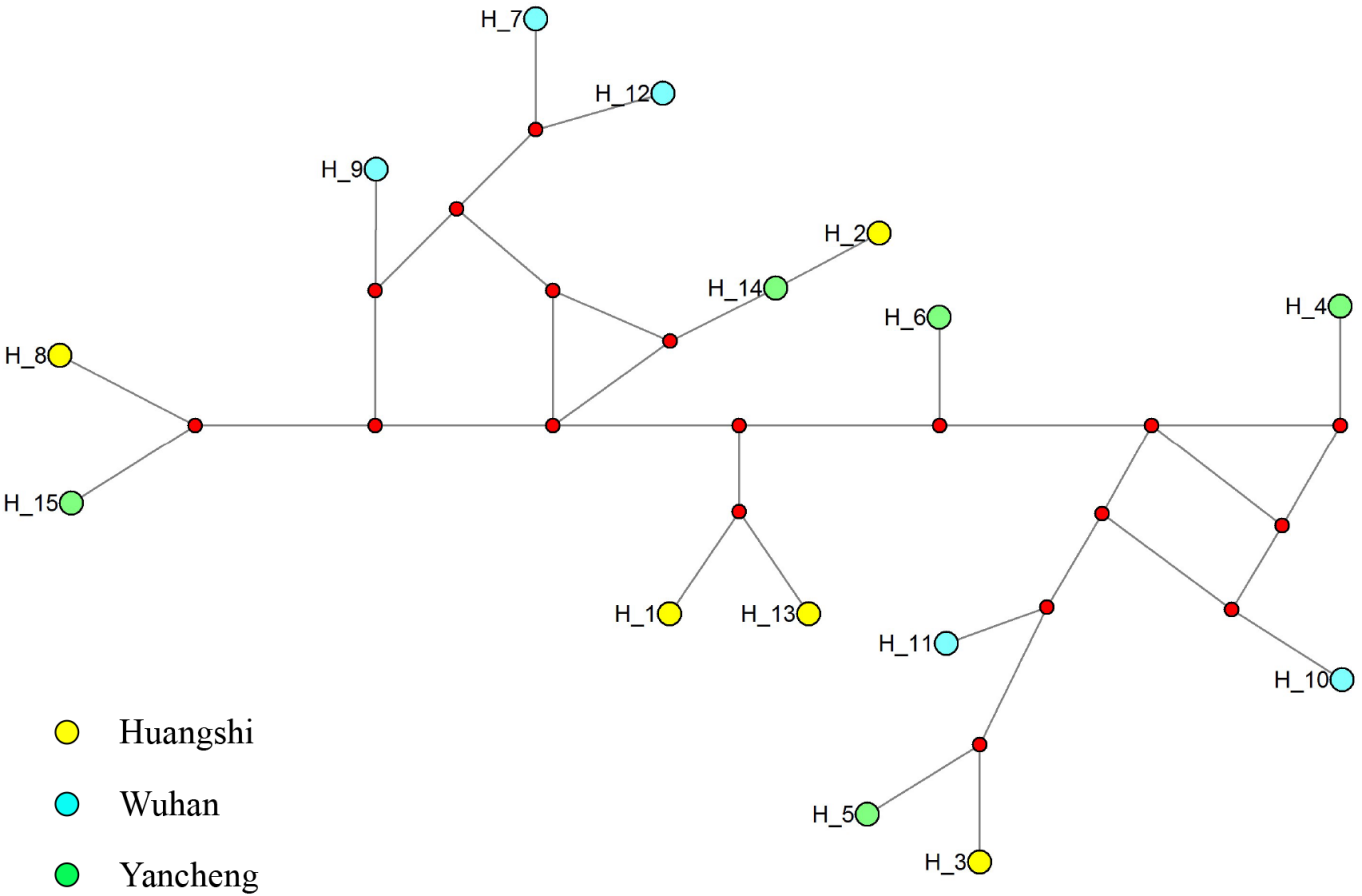
Median-joining haplotype network of Rpb1 sequences from *Neoflabelliforma dubium* n. sp. isolates. Yellow, blue and green circles represent haplotypes obtained from different locations (Huangshi, Wuhan and Yancheng, respectively). Red circle indicates median vectors.

To further explore the genetic diversity of the present species, Rpb1 gene sequences were successfully amplified for all 3 isolates, and the obtained sequences were deposited in GenBank under accession numbers OP852430-OP852444. Five sequences per isolate were obtained, with 15 sequences totally. The alignment of the 15 sequences revealed 89 polymorphic sites, among which included 88 transitions and 10 deletions (**Figure 5**). To explore the relationship between sequences and isolates, a haplotype network was constructed. Results clearly indicated that haplotypes isolated from the same isolates did not cluster together (**Figure 6**). The Rpb1 gene displayed high level of synonymous variation. The pooled pairwise nucleotide diversity at synonymous sites (*π*_s_) was 8.94 (**Table 4**). Differences among different populations were small, indicating that there are no differences of genetic variation among *N. dubium* n. sp. populations. The pooled *π*_A_ value was 0.47, twenty times lower than that observed at synonymous sites.

**Table 4:** Nucleotide diversity, Tajima’s D, and Fu’s Fs based on Rpb1 of *Neoflabelliforma dubium* n. sp.

Origin	N	Synonymous			Non-synonymous			Fu’s Fs
		*π*_s_	*θ*_WS_	*D*_S_	*π*_A_	*θ*_WA_	*D*_A_	
HS	5	9.28	9.19	-0.38	0.51	0.58	-0.89	0.78
WH	5	8.89	NA	0.03	0.51	NA	-1.16	0.65
YC	5	7.42	NA	-0.18	0.63	NA	-0.95	0.53
Total	15	8.94	NA	0.14	0.47	NA	-1.85*	0.65

N, number of sequences; HS, Huangshi, WH, Wuhan, YC, Yancheng; π_s_ and π_A_, pairwise nucleotide diversity at synonymous and nonsynonymous sites expressed as percentage, respectively; θ_WS_ and θ_WA_, nucleotide site variability based on the number of synonymous and nonsynonymous segregating sites expressed as percentage, respectively; D_S_ and D_A_, Tajima’s D at synonymous and nonsynonymous sites, respectively; NA, not available; statistical significance of Tajima´s D, *P<0.05.

**Figure 7:**
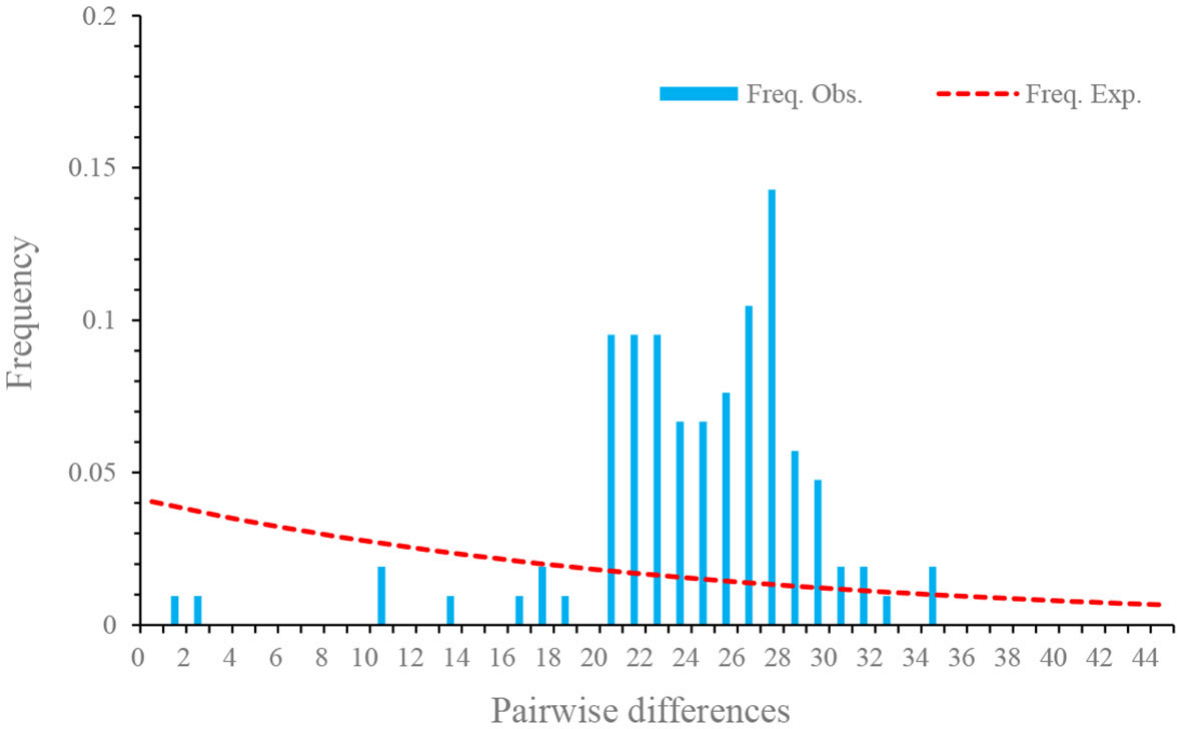
Mismatch distributions of *Neoflabelliforma dubium* n. sp. population based on the Rpb1 gene.

The pooled *D* at synonymous sites (*D*_S_) was 0.14. The estimates of *D* at synonymous sites (*D*_S_) of different isolates were different, but no significant difference was observed among different populations. The pooled *D* at non-synonymous sites (*D*_A_) was -1.85 (p < 0.05), suggesting an excess of low frequency mutations (**Table 4**). D values at nonsynonymous sites of different isolates were also negative, but no statistically significant differences were observed. Fu’s F neutrality test was positive for the different isolates, indicating that the population expansion may not occur (**Table 4**). In order to further explore the possible population expansion, we performed a mismatch analysis. Result indicated that the population showed a multi-peak model rather than a single peak, demonstrating that there is no population expansion for *N. dubium* n. sp. (**Figure 7**).

The overall F_st_ and Nm of all isolates were 0.06974 and 6.67, respectively, suggesting low genetic differentiation and high gene flow of *Neoflabelliforma dubium* n. sp. among different populations. Hangshi isolates and Yancheng isolates had the highest Fst (0.14781), while Hangshi isolates and Wuhan isolates had the lowest Fst (-0.00553) (**Table 5**). The gene flow was the largest among Wuhan and Yancheng isolates (9.93) and the smallest among Wuhan and Huangshi isolates (-90.96) (**Table 5**).

**Table 5:** Pairwise genetic differentiation (Fst: left bottom) and gene flow (Nm: upper right) among *Neoflabelliforma dubium* n. sp. isolates based on Rpb1 gene.

Region	HS	WH	YC
HS	–	-90.96	2.88
WH	-0.00553	–	9.93
YC	0.14781	0.05639	–

HS, Huangshi, WH, Wuhan, YC, Yancheng.

The analysis of molecular variance showed that the major variance occurred within isolates, up to 93% of the variance. Whilst, the difference among isolates was not significant, which accounted for 7% of the total variance (**Table 6**).

**Table 6:** Analysis of molecular variance (AMOVA) of *Neoflabelliforma dubium* n. sp. based on Rpb1 gene.

Source of variation	d.f.	SS	VC	% var	P
Among isolates	2	30.93	0.84	6.97	ns
Within isolates	12	135.00	11.25	93.03	ns
Total	14	165.93	12.09		

d.f., degrees of freedom; SS, sum of squares; VC, variance components; % var, percentage of variation; P, probability of a random variance component value ≤ observed value; ns, non-significant.

**Figure 8:**
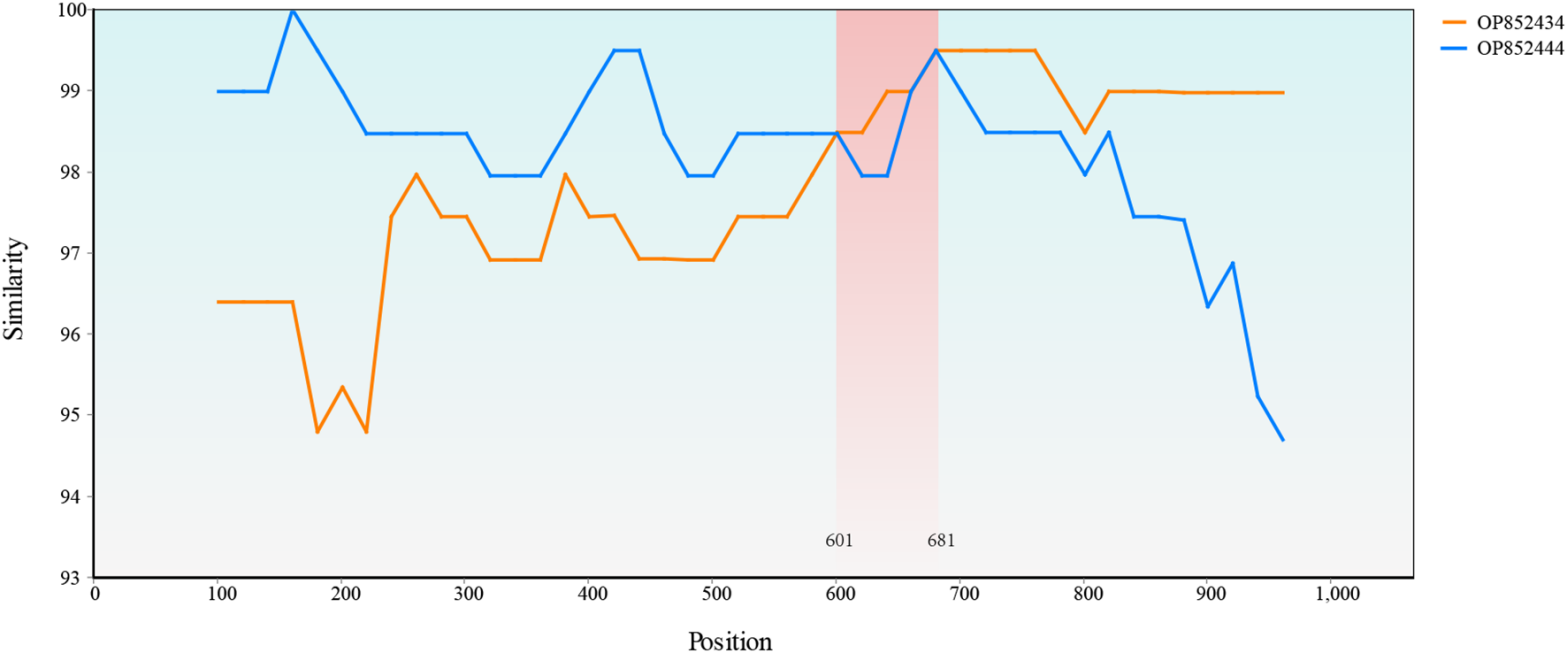
Simplot evidence for recombination event. Recombination analysis of the OP852439. The recombinant breakpoints were located at 601 bp and 681 bp.

Only one recombination event supported by the SiScan and Chimaera as well as 3Seq methods was detected with RDP4. A recombination event that occurred between OP852434 and OP852444 led to the generation of recombinant OP852439. A SimPlot of this recombination event is presented in **Figure 8**, in which the sequences OP852444 and OP852434 were used as the major and minor parental sequences, respectively.

**Taxonomic summary**

Name: *Neoflabelliforma dubium* n. sp.

Genus: *Neoflabelliforma*

Type host species: *Diaphanosoma dubium* (Crustacea: Sididae)

Type locality: Eutrophic ponds of Huangshi city, Hubei province, China (30° 17^′^46.49^′′^ N, 114° 44^′^8.53^′′^ E), Jinyin Lake of Wuhan city, Hubei province (30° 38^′^21.35^′′^ N, 114° 11^′^28.64^′′^ E), and Yanlong Lake of Yancheng city, Jiangsu province, China (33° 20^′^0.43^′′^ N, 120° 1^′^39.65^′′^ E).

Site of infection: Adipose tissue.

Sporogony: Uninucleate sporont was not observed, multinucleate sporogonial plasmodia with isolated nuclei. Sporoblasts are formed by rosette-like budding of sporogonial plasmodia.

Spore: Oval spores are uninucleate, 4.02 ± 0.24 (3.63-4.53) µm long and 2.27 ± 0.15 (2.12-2.57) µm wide. Isofilar polar filaments coil 13-17 turns and arrange in 2-3 rows. The polaroplast is bipartite with narrow anterior lamellae and loose posterior lamellae. The spore wall consists of a 50-59 nm thick electron-dense exospore and a 97-132 nm thick electron-lucent endospore. The exospore consisted of two layers, including the electron-moderate layer and electron-dense layer of tubular projections.

Type material: Syntype specimens of TEM resin blocks were deposited in the Museum of Hydrobiological Sciences, Institute of Hydrobiology, Chinese Academy of Sciences with accession number of MTR20221001.

Etymology: The species name relates to host species name.

Gene sequences: Depositing in GenBank under accession numbers of OP859151-OP859153, OP881373-OP881375 and OP852430-OP852444.

## 4 Discussion

The morphological features of the novel microsporidium are similar to the diagnostic characteristics of the genus *Neoflabelliforma*, isolated nuclei in life stages, isofilar polar filament arranging in several rows in the middle of the spore and one row at posterior, multilayered exospore, lamellar polaroplast, and the precursor of exospore associating with the dense tubular secretions on the surface of sporogonial plasmodia (Morris and Freeman, 2010). Only one *Neoflabelliforma* species has been reported previously, i.e. *N. aurantiae* infecting the *Tubifex tubifex* and hyperparasitising the concurrent Aurantiactinomyxon-type myxosporean released from *Tubifex tubifex* (Morris and Freeman, 2010). The morphological comparison between the present species with *N. aurantiae* is summarized in **Table 7**. N. *dubium* n. sp. can be easily differentiated from *N. aurantiae* by its greater size (4.02 × 2.26 vs. 3.4 × 1.9), different number of polar filament coils (9-11 vs. 15), and different number of exospores layers (2 vs. 3). Moreover, *N. dubium* n. sp. possesses a bipartite polaroplast with tightly packed anterior lamellae and a loosely aligned posterior lamellae, rather than packed lamellar polaroplast, as in the case of *N. aurantiae.* In addition, the development of *N. dubium* n. sp. was in direct contact with the host cell cytoplasm, rather than within sporophorous vesicles which was the one of the diagnostic characteristics of the genus *Neoflabelliforma*. Similar phenomenon has been found in the genus *Liebermannia* (Sokolova et al., 2009), *Neoperezia* (Issi et al., 2012) and *Ovipleistophora* (Weng et al., 2021b), suggesting that presence of sporophorous vesicles referred from the type species maybe not be the reliable taxonomic criterion of these genera due to morphological plasticity.

**Table 7:** Morphological comparison of *Neoflabelliforma dubium* n. sp. with *Neoflabelliforma aurantiae*.

Characters	*Neoflabelliforma dubium* n. sp.	*Neoflabelliforma aurantiae*
Host	*Diaphanosoma dubium*	*Tubifex tubifex* Aurantiactinomyxon-type myxosporean
Infected site	Adipose tissue	Various tissue of oligochaete, binucleate cells of myxosporean
Spore shape	Ovoid	Ovoid
Spore size (µm)	4.02 × 2.26	3.4 × 1.9
Polar filament type, number	Isofilar, 9-11 coils	Isofilar, 15 coils
Polaroplast	Closely packed anterior and wider posterior lamellae	Closely packed lamellae
Exospore	Two-layered	Three-layered
Parasite-host interface	Direct contact with host cell cytoplasm	Presence of sporophorous vesicle
References	(Herein)	(Morris and Freeman, 2010)

The average levels of synonymous diversity (*π*_s_) of *N. dubium* n. sp. were about 8% at the Rpb1 gene, which was much higher than all previously reported species, such as *Vairimorpha apis* (1.68%) and *V. ceranae* (1.58%) (Maside et al., 2015; Tokarev et al., 2020), suggesting that *N. dubium* n. sp. possesses substantial variation at this locus. Compared to high level diversity at synonymous sites, non-synonymous variation was much lower. The similar findings were previously reported in some invertebrates-infecting species based on the same molecular marker, such as *V. apis*, *V. ceranae* (Maside et al., 2015; Tokarev et al., 2020), and *N. bombycis* (Ironside, 2013). These results demonstrated that amino acid mutations can be removed in these species, revealing that Rpb1 gene is maintained by purifying selection.

Though the *D*_S_ values among different populations were not significantly negative, the pooled *D*_S_ value reached a statistically significant negative, reflecting that *N. dubium* n. sp. may have undergone recent population expansion, as previously reported in *V. ceranae* (Maside et al., 2015; Tokarev et al., 2020). However, Fu’s F_S_ test produced a positive value, which means that *N. dubium* n. sp. has not experienced population expansion. The lack of population expansion is also demonstrated by the multi-peak model of the Mismatch analysis. Taken together, it can be concluded that no population expansion occurred in *N. dubium* n. sp. Based on the present result, we found that Fu’s F_S_ is a more sensitive indicator of population expansion than Tajima’s D, which is consistent with previous report in *V. ceranae* based on the multi-loci sequences analysis (Roudel et al., 2013; Tokarev et al., 2020).

The low Fst indices and high Nm suggested the lack of population divergence among *N. dubium* n. sp. isolates from different locations. Moreover, the analysis of molecular variance revealed that the major variance occurred within isolates, rather than among isolates, which further reinforced this finding on population relatedness. A similar finding was previously reported in *V. ceranae* based on multiple molecular markers, such as PTP2, PTP3 and RPB1 (Gomez-Moracho et al., 2015; Maside et al., 2015; Tokarev et al., 2020). The recent worldwide expansion is the reason to explain the lack of genetic divergence of *N. ceranae*. Frequent gene flow among there studied isolates of the novel species from the same watershed can partially explain why there is no population divergence among *N. dubium* n. sp. isolates.

Interestingly, the genetic recombination was detected in *N. dubium* n. sp., suggesting that *N. dubium* n. sp. may undergo a meiotic phase in the life cycle. However, ultrastructural observations showed that isolated nuclei were found throughout the sporogony of *N. dubium* n. sp., and no diplokaryotic cells were observed, indicating that the meiotic process was not proven by cytological observations. Similar results have also been reported in some daphnia-infecting microsporidian species, such as *Berwaldia schaefernai* (Gonzalez-Tortuero et al., 2016) and *Hamiltosporidium magnivora* (Haag et al., 2013). The presence of meiotic spores in the secondary host may be the possible reason for the lack of the meiotic stage in daphnia hosts. The presence of meiosis and sexual reproduction has been reported in the definitive hosts of microsporidian species with multi-host lifecycles, such as *Amblyospora* spp. (Sweeney et al., 1990) and *Hyalinocysta* spp. (Andreadis and Vossbrinck, 2002). *N. aurantiae*, the type species of the genus *Neoflabelliforma*, which infects the oligochaete *Tubifex tubifex* and Aurantiactinomyxon-type myxosporean (Morris and Freeman, 2010). Thus the infection of *N. dubium* n. sp. in the daphnid *Diaphanosoma dubium* extended the host range of *Neoflabelliforma* species, suggesting that *Neoflabelliforma* spp. possibly have a multi-host lifecycle. Therefore, further studies are required to disclose the lifecycle of the *Neoflabelliforma* species. Studying the genetic diversity of populations is essential for understanding how species evolve. Although high genetic diversity has been reported in Microsporidia, especially for some insect-infecting species (Gomez-Moracho et al., 2015; Hassan et al., 2020), little is known about the genetic diversity in aquatic microsporidian species. In the present study, we proved the high level of genetic diversity of Rpb1 sequences of *N. dubium* n. sp. isolates, which is coincident with previously reported species, such as *Vairimorpha apis* (Maside et al., 2015), *V. ceranae* (Gomez-Moracho et al., 2014; Tokarev et al., 2020) and *N. bombycis* (Ironside, 2013). However, the mechanisms underlying the presence of high genetic diversity of the single copy gene within isolates remain unknown. The presence of diploidy or polyploidy in these species was one of the possible factors to explain it. Pelin et al. (2015) found that the high genetic diversity within isolates resulted from the presence of polyploidy in *V. ceranae*. In addition, Watson et al. (2015) found that high level of single nucleotide polymorphism is because *Trachipleistophora hominis* is diploid at some stage of its lifecycle. Though the developmental stages of *N. dubium* n. sp. are uninucleate, several unikaryotic microsporidia has been proved to be diploid, such as *Hamiltosporidium tvaerminnensis* (Haag et al., 2013), *Nematocida parisii* (Cuomo et al., 2012), and *Encephalitozoon cuniculi* (Selman et al., 2013). The occurrence of recombination in *N. dubium* n. sp. further proves its possible diploidy or polyploidy. Therefore, the high genetic diversity in *N. dubium* n. sp. isolates could be due to the presence of diploid nuclei and recombination events.

In terms of the phylogenetic position of the genus *Neoflabelliforma*, the previous analysis indicated that *Neoflabelliforma aurantiae* formed a solitary branch between the identified clades (Dubuffet et al., 2021; Park and Poulin, 2021). Recent phylogenetic analysis showed that *N. aurantiae* clustered firstly with *Hamiltosporidium magnivora*, and then formed a sister group with a late evolutionary branch consisting of *Astathelohania contejeani* and *Areospora rohanae*, which collectively formed an independent branch (Bojko et al., 2022). So, the natural phylogenetic position of *Neoflabelliforma* spp. remains unresolved. The present phylogenetic analysis indicated that two available *Neoflabelliforma* species clustered with *Microsporidium* sp. isolated from the soil to form a solitary basal branch of clade 4 defined by Vossbrinck and Debrunner-Vossbrinck (2005), which was consistent with the previous report (Ardila-Garcia et al., 2013). So, our result supports that that the genus *Neoflabelliforma* is monophyletic. Given the low sequence similarity (less than 80%) between *Neoflabelliforma* spp. and the reported microsporidian species, and the closest sequence of *Neoflabelliforma* spp. isolated from the soil sampled in the Pacific Northwest, it can be suspected that high diversity of microsporidian species in this branch waits to be uncovered. Taken together, considering the special phylogenetic position of the genus *Neoflabelliforma* in the microsporidian systematics and high diversity of aquatic microsporidia, it can be supposed that *Neoflabelliforma* spp. could represent an independent clade among the microsporidian taxonomy.

Ribosomal DNA genes have been widely used for species identification and phylogenetic reconstruction of the Microsporidia for it is highly conserved. However, recent research showed that the ribosomal DNA genes are highly variable in some microsporidian species (O'Mahony et al., 2007; Sagastume et al., 2011; Liu et al., 2013). In the present study, we found that the rDNA (including SSU, ITS and LSU) sequences are highly conserved in all three *N. dubium* n. sp. isolates, which are similar to some daphnid-infecting microsporidian species (Vávra et al., 2018), rather than some insect-infecting microsporidian species (Sagastume et al., 2011; Liu et al., 2013). The conservative rDNA may be the result of the concerted evolution for these microsporidia species (Ironside, 2013). These results indicate that SSU, ITS and LSU rDNA are suitable molecular markers for the identification of *N. dubium* n. sp.

In summary, we characterized a novel microsporidian species, nominated as *Neoflabelliforma dubium* n. sp. from the adipose tissue of *Diaphanosoma dubium* in the middle and lower reaches of the Yangtze River. Genetic analysis referring from Rpb1 sequences indicated that sexual reproduction possibly occurred in *N. dubium* n. sp., and its life cycle possible involve several hosts.

## Data availability statement

The datasets presented in this study can be found in online repositories. The names of the repository/repositories and accession number(s) can be found below: https://www.ncbi.nlm.nih.gov/genbank/, OP859151-OP859153; https://www.ncbi.nlm.nih.gov/genbank/, OP881373-OP881375; https://www.ncbi.nlm.nih.gov/genbank/, OP852430-OP852444.

## Author contributions

MW and XZ collected samples, performed parasitological examination, data analysis and helped write the manuscript; ZX, SX, QZ, and AL performed morphological comparisons and helped write the manuscript. JZ designed this study and drafted the manuscript. All authors contributed to the article and approved the submitted version.
